# Outlook of Cell Gene Therapies Development and Approval from Quality and Regulatory Perspective

**DOI:** 10.1007/s43441-026-00920-4

**Published:** 2026-02-12

**Authors:** Carolina Iglesias-Lopez, Antonio Vallano, Antònia Agustí

**Affiliations:** 1 Gilead Sciences, Dublin, Ireland; 2 Pharmacology, Therapeutics and Toxicology Department, Universitat Autònoma de Barcelona, Barcelona, Spain; 3 Medicines Area, Catalan Health Service (CatSalut), Barcelona, Spain; 4 Catalan Institute of Health (ICS), Barcelona, Spain; 5 Clinical Pharmacology Service, Vall Hebron University Hospital, Barcelona, Spain

**Keywords:** Cell gene therapies, Advanced therapy medicinal products, Regulatory, Quality, Marketing authorization

## Abstract

**Background:**

Cell and gene therapies represent a transformative advance in modern medicine but pose major quality and regulatory challenges. The complexity of manufacturing, product comparability, and potency assessment often limits dossier robustness and delays approval.

**Methods:**

A systematic review was conducted on cell gene therapies approved in the European Union (EU) and the United States (US) up to December 2024. Publicly available regulatory data from EMA and FDA sources were analyzed to identify key regulatory milestones and quality issues during marketing authorization.

**Results:**

Fourteen cell gene therapies were approved (12 in the US, 11 in the EU). All received orphan designation, and over 80% benefited from expedited development pathways. The most frequent *quality objections* concerned manufacturing comparability, potency assay validation, specifications, and stability data. Although regulatory support mechanisms accelerated submissions, they did not consistently translate into higher-quality dossiers. The FDA follows a data-driven approach, while the EMA takes a broader, science-based view and continuous improvement.

**Conclusion:**

Ensuring robust quality data packages remains the main bottleneck in cell gene therapy development. Early integration of quality-by-design principles, comprehensive comparability assessments, and validated potency assays are essential to strengthen regulatory submissions. Continuous dialogue with regulatory agencies and harmonization between regions are key to accelerating patient access while maintaining product quality and consistency.

**Supplementary Information:**

The online version contains supplementary material available at https://doi.org/10.1007/s43441-026-00920-4.

## Introduction

Cell gene medicinal products are a specific type of advanced therapy medicinal products (ATMPs) that fall within the broader category of gene therapies. Unlike other gene therapies that deliver genetic material directly into the patient’s body (in vivo approaches), cell gene medicinal products have involved so far ex vivo genetic modification of patient-derived cells before reintroducing them into the body [1]. Most of these therapies are currently autologous, meaning they are individually manufactured for each patient by extracting their own cells and genetically modifying them using biological tools such as viral vectors or CRISPR/Cas technology [2, 3]. So far, cell gene medicinal products have represented one of the major advancements in different therapeutic areas including malignancies, immune-mediated disorders and other hereditary genetic disorders, offering highly personalized and targeted treatment strategies. Chimeric Antigen Receptor (CAR)-based cell therapies in hematology has lead and remained the primary clinical focus for cell therapies, but cell therapies in treating autoimmune diseases is growing rapidly [4, 5]. The goal of these therapies is to be safer and more effective than traditional treatments with the aim of becoming the new standard of care in the coming years or the cure for certain diseases [6]. These medicinal products are complex biological medicinal products, subject to composite regulatory framework and several development challenges are to be resolved yet for these therapies to be commonly used in the clinical setting [7].

This review analyzes the current state of cell gene therapies from a regulatory and quality development perspective in both the European Union (EU) and the United States (US). It highlights key features and limitations impacting the approval process for these innovative therapies and provides insights for developers to anticipate potential issues during the application review from a quality and regulatory standpoint.

## Methods

A systematic review of the development features that supported the marketing authorisation (MA) of the ATMPs approved in the US and EU was carried out using the following approach:

(i) *Search* strategy: Data collection was primarily extracted from the Food and Drug Administration (FDA) and the European Medicines Agency (EMA) websites (www.fda.gov; www.ema.europa.eu). European data was gathered from European Public Assessment Reports, orphan designations product reports and publicly available EMA agendas, minutes and highlights. The US data were collected mainly from FDA drug summaries reports and other approval history related documents published for the approved cellular and gene therapy products. The search was carried out until December 2024.
(ii) *Eligibility criteria*: Only cell gene therapy products classified as ATMPs according to the EMA criteria [8] and US criteria [9]. For the EU products, only those authorised under centralised procedure have been considered. Other ATMPs products consisting of gene therapies such as adeno-associated virus (AAV) vector-based gene therapy, cell therapies (without being genetically modified), tissue engineered products or HPC Cord Blood products (US) are not included in the analysis, i.e., ChondroCelect, MACI, Holoclar, Spherox, Glybera, Zalmoxis, Alofisel, Ebvallo, Glybera, Imlygic, Luxturna, Zolgensma, Roctavian, Uptaza, Hemgenix, Beqvez (Durveqtix), Adstiladrin, Laviv, Gintuit, Kebilidi, Rethymic, Symvess, Stratagraf, Vyjuvek (approved ATMPs until Dec 2024).
(iii) *Data extraction and collected variables*: The authors designed specific data extraction forms using Excel 2019 (Microsoft Corporation, Redmond, WA, USA) to collect information.

- *Regulatory development—*The pharmacotherapeutic group has been classified according to the ATC system. Time for approval in the EU was considered from the application was received by the EMA until CHMP Opinion. Time for approval in the US was considered from the date of Biological License Application (BLA) submission to the date of publication of approval letter. For those products with rolling review, the BLA submission is considered when the final module of rolling BLA was received. Only the initial Marketing Authorization Applications (MAA) (EU) or BLA (US) have been considered, not including subsequent variations (EU) or supplements (US).
- *Quality development—*European public assessment reports (EPARs) have been analyzed identifying the major concerns and other concerns. The major concerns and other concerns are defined in the EPARs of each product and were not subject to the author’s classification. Each of the issues were classified according to the following criteria in the EU: vector manufacturing, overall manufacturing process and process controls, process validation, stability data, potency assay related, cell transduction, microbiological control, release strategy, comparability assessment for manufacturing processes and others. Each of the issues were classified according to the following criteria in the US: stability, container closure system, reference standards or materials, characterization of impurities, batch analysis, analytical procedures and validation of analytical procedures, specification(s) and justification of specifications(s), control of excipients, process validation and/or evaluation, control of critical steps and intermediates, description of manufacturing process, chain of identity, manufacturer(s), compatibility, microbiological attributes, manufacturing process development, formulation development, impurities and control of materials. This analysis captures the deficiencies/issues with the content of the first application for both regions, considering that the applicant might submit an application to one regulatory authority and subsequently to another with an update to the dossier between these two submissions. The agency documents do not disclose all data, therefore only approval issues could be drawn on the disclosed data.

(iv) Statistical analysis: A descriptive statistical analysis was performed on the collected data. Measures of central tendency (median), quartiles 25 and 75 (Q25, Q75) and frequency distributions (percentages) were used to summarize the regulatory timelines, designations, and quality-related issues. No inferential statistics were applied due to the limited sample size and the descriptive nature of the study. Microsoft Excel 2019 (Microsoft Corporation, Redmond, WA, USA) was used for data analysis and visualization.

## Results

Until December 2024, there are 28 approved ATMPs in the EU and 34 in the US, 14 pertaining to cell gene therapies (35–39%); 12 products approved by the Food and Drug Administration (FDA) and/or 11 products approved by the European Medicines Agency (EMA) (Table 1).

**Table 1:** Approved cell gene therapies summary

Trade name	Active ingredient	Genetic modification method	Vector type	EMA authorized indication / EMA authorization type	FDA authorized indication/ FDA authorization type
Strimvelis^®^	Ex vivo gene modified cells	Gene addition	Retroviral	Severe combined immunodeficiency due to adenosine deaminase deficiency (ADA-SCID)/conditional approval	No FDA approved indication
Kymriah^®^	Tisagenlecleucel	CAR-T	Lentiviral	Relapsed or refractory B-cell precursor acute lymphoblastic leukemia (ALL), diffuse large B-cell lymphoma (DLBCL)/standard approval	Relapsed or refractory B-cell precursor acute lymphoblastic leukemia (ALL), large B-cell lymphoma (LBCL), follicular lymphoma (FL) / standard Approval
Yescarta^®^	Axicabtagene ciloleucel	CAR-T	Retroviral	Relapsed or refractory diffuse large B-cell lymphoma (DLBCL) and primary mediastinal large B-cell lymphoma (PMBCL)/standard approval	Relapsed or refractory large B-cell lymphoma after two or more lines of systemic therapy, follicular lymphoma (FL)/standard approval
Zynteglo^®^	Betibeglogene autotemcel	Gene addition	Lentiviral	Transfusion-dependent beta-thalassemia (TDT)/conditional approval	No longer marketed in the US
Libmeldy^®^ Lenmeldy^®^	Atidarsagene autotemcel	Gene addition	Lentiviral	Metachromatic leukodystrophy (MLD)/conditional approval	Metachromatic leukodystrophy (MLD)/standard approval
Tecartus^®^	Brexucabtagene autoleucel	CAR-T	Retroviral	Relapsed or refractory mantle cell lymphoma (MCL), Relapsed or refractory B-cell precursor acute lymphoblastic leukemia (ALL)/standard approval	Relapsed or refractory mantle cell lymphoma (MCL), Relapsed or refractory B-cell precursor acute lymphoblastic leukemia (ALL)/standard approval
Breyanzi^®^	Lisocabtagene maraleucel	CAR-T	Lentiviral	Relapsed or refractory diffuse large B-cell lymphoma (DLBCL), high-grade B-cell lymphoma (HGBCL), primary mediastinal large B-cell lymphoma (PMBCL), and follicular lymphoma grade 3B/standard approval	Relapsed or refractory large B-cell lymphoma (LBCL) after two or more lines of systemic therapy, follicular lymphoma (FL)/standard approval
Abecma^®^	Idecabtagene vicleucel	CAR-T	Lentiviral	Relapsed and refractory multiple myeloma/standard approval	Relapsed and refractory multiple myeloma/standard approval
Skysona^®^	Elivaldogene autotemcel	Gene addition	Lentiviral	Cerebral adrenoleukodystrophy (CALD)/conditional approval	Cerebral adrenoleukodystrophy (CALD)/standard approval
Carvykti^®^	Ciltacabtagene autoleucel	CAR-T	Lentiviral	Relapsed and refractory multiple myeloma/standard approval	Relapsed and refractory multiple myeloma/standard approval
Casgevy^®^	Exagamglogene autotemcel	Genome editing (CRISPR-Cas9)	N/A	Severe sickle cell disease and transfusion-dependent beta-thalassemia (TDT)/conditional approval	Severe sickle cell disease/standard approval
Lyfgenia^®^	Lovotibeglogene autotemcel	Gene addition	Lentiviral	No EMA approved indication	Sickle cell disease/standard approval
Aucatzyl^®^	Nimacogene vepocogene	CAR-T	Lentiviral	No EMA approved indication	Deficiency in the enzyme adenosine deaminase (ADA-SCID) in patients for whom no suitable human leukocyte antigen (HLA)-matched related stem cell donor is available. Deficiency in the enzyme adenosine deaminase (ADA-SCID) in patients for whom no suitable human leukocyte antigen (HLA)-matched related stem cell donor is available/standard approval
Telcera^®^	Lenadogene noltemcel	TCR	Lentiviral	No EMA approved indication	Deficiency in the enzyme adenosine deaminase (ADA-SCID) in patients for whom no suitable human leukocyte antigen (HLA)-matched related stem cell donor is available/standard approval

CAR-T, chimeric antigen receptor T-cell; TCR, T cell receptor-Cell; N/A, Not applicable

There are currently two main groups of cell gene therapies: autologous CAR/TCR-based cell therapies and autologous hematopoietic stem cell-based gene therapies. The pharmacotherapeutic group include “L01 Antineoplastic agents” (57.1%, *n* = 8/14), “B06A Other hematological agents” (21.4%, *n* = 3/14), “N07 Other nervous system drugs” (14.3%, *n* = 2/14) and “L03 Immunostimulants” (7.1%, *n* = 1/14).

These therapies can be grouped into: CD19-directed genetically modified autologous T cells (5 products), B-cell maturation antigen (BCMA)-directed genetically modified autologous T cells (2 products), T cell receptor (TCR)-directed genetically modified autologous T cells (1 product) and autologous hematopoietic stem cell-based gene therapy (6 products). 

### Regulatory Development

**Fig. 1:**
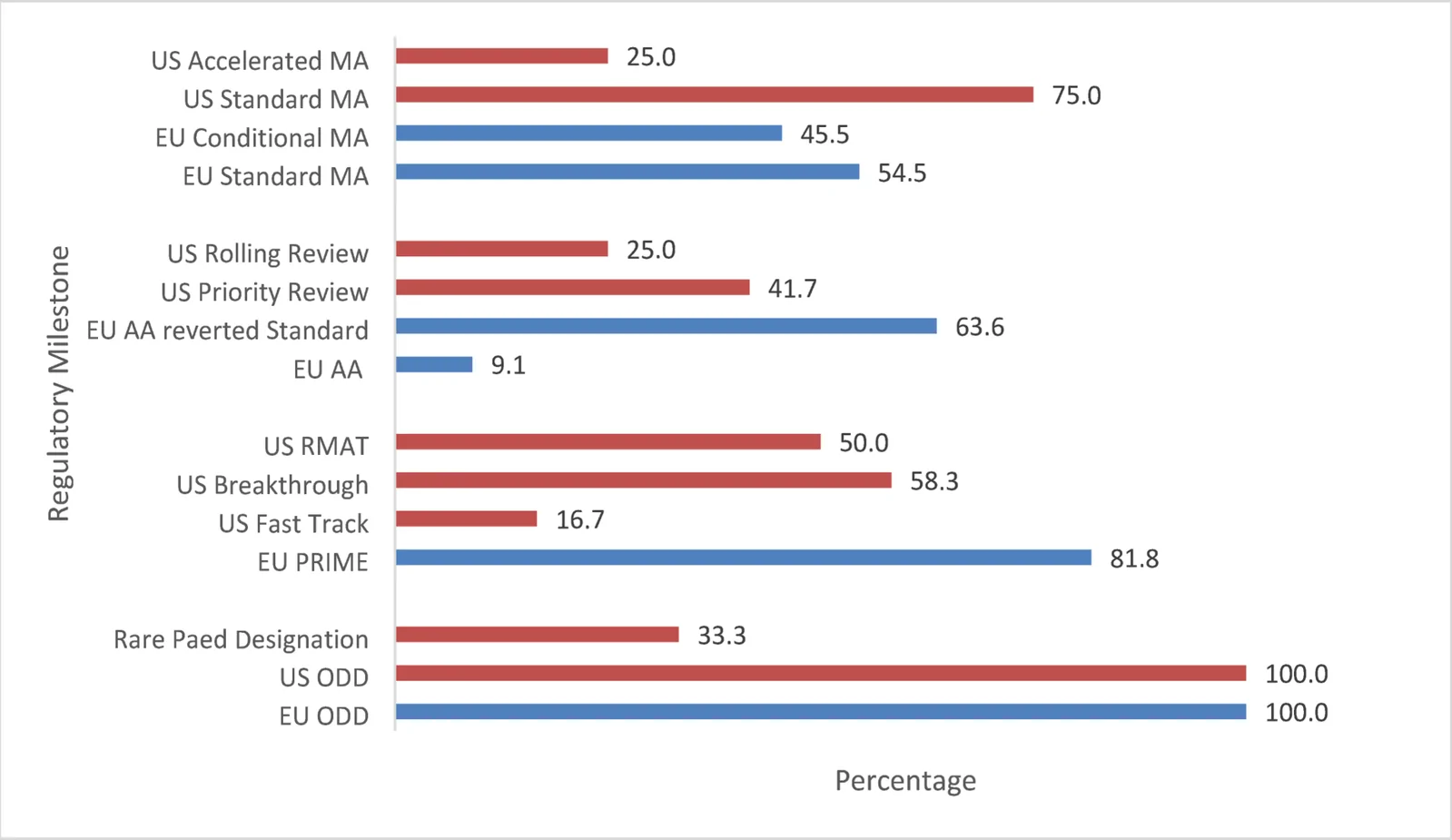
Regulatory milestones for approved cell gene therapies in the EU and the US. AA, accelerated assessment; BLA, US biological license application; ODD, orphan drug designation; MA, marketing authorization; MAA, marketing authorization application; PRIME, priority medicines EMA scheme; RMAT, regenerative medicine advanced therapy designation

The key regulatory milestones for these approved products in both regions are shown in Fig. 1. All these approvals were granted with an orphan drug designation (ODD) during their development in both regions, and 4 out of 5 products developed for pediatric indications obtained the US Rare Pediatric Designation (from total of *n* = 12). Almost all these therapies (*n* = 13) had expedited development designation, such as EU PRIME and/or US RMAT/Breakthrough/Fast Track designations. The MAA review was also expedited through an accelerated assessment (AA) in the EU (*n* = 8/11), where *n* = 7/8 of the products were reverted this AA to standard MAA timelines. In the US, the priority review was granted for *n* = 5/12 of the applications and the rolling review for *n* = 3/12 of the products.

**Fig. 2:**
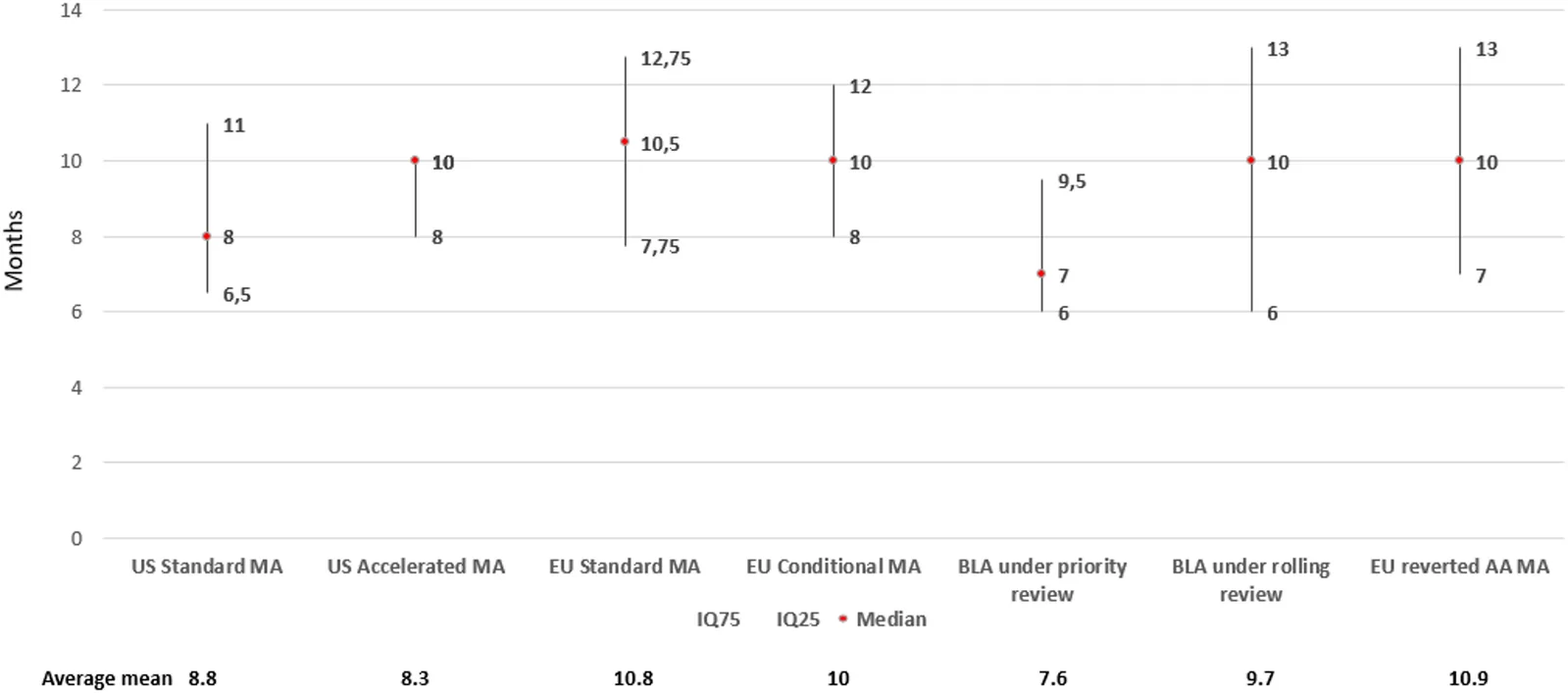
Time from MAA/BLA submission to approval for approved cell gene therapies in the EU and the US. AA, accelerated assessment; BLA, US biological license application; MA, marketing authorization; MAA, marketing authorization application

The overall median time (IQR 25–75) from submission to approval is around 10 (8–11) months in the EU and 8 (7–10.7) months in the US. The US priority review implied 7.6 months of BLA procedure. The rolling review did not reduce the time of BLA procedure with respect to the standard BLA. Most of the approvals in the US consisted of standard MA (*n* = 9/12), while in the EU, *n* = 6/11 were standard MAs and *n* = 5/11 consisted of conditional approvals. The type of MA did not have a significant impact on the time for MAA and BLA procedures (Fig. 2).

### Quality Development

So far, all approved cell gene therapies are autologous and genetically modified ex-vivo. Most of these therapies are transduced using a lentiviral vector encoding for the gene of interest (*n* = 10/14), followed by retroviral vector (*n* = 3/14) or by using CRISPR-Cas9 genome editing tool (*n* = 1/14).

**Fig. 3:**
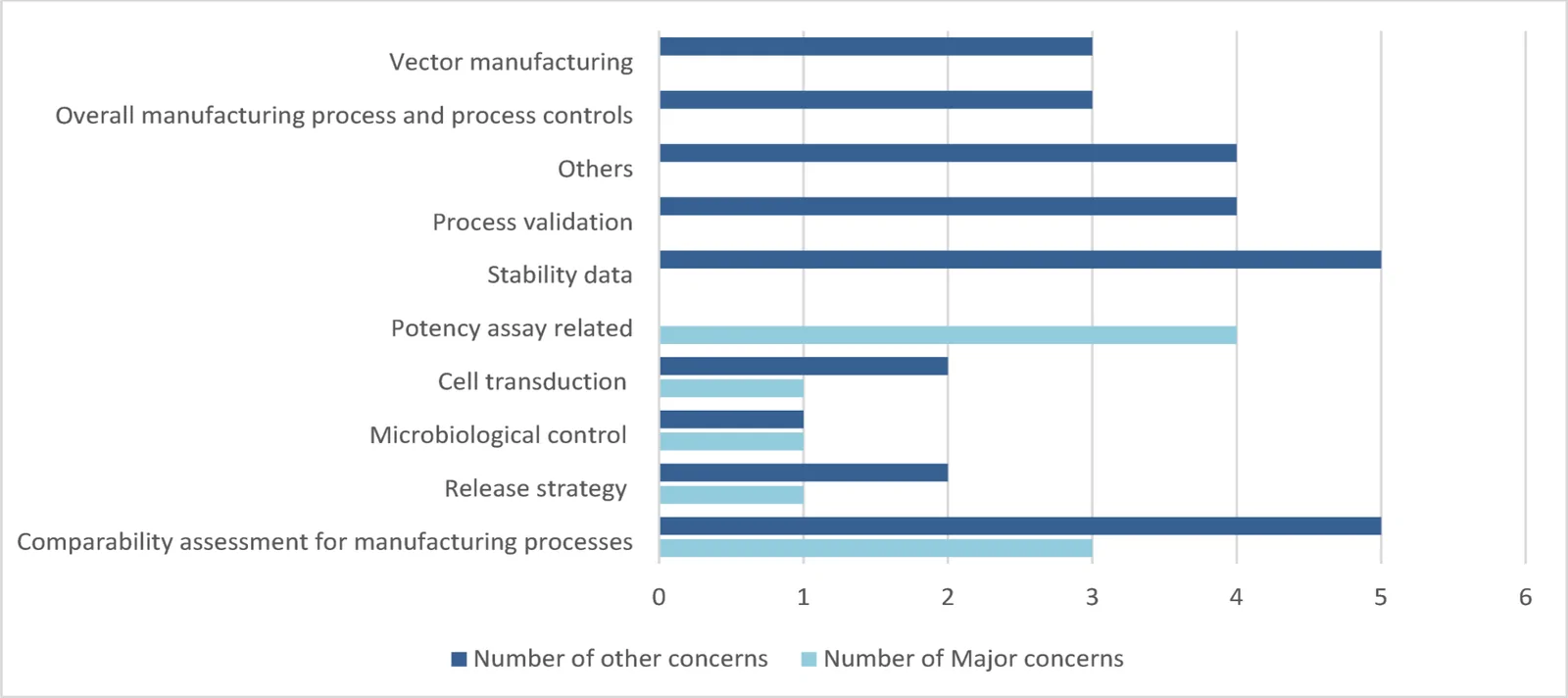
EU quality major objections and other issues found in the marketing authorization application for approved cell gene therapies (Total number of concerns considering all applications analyzed)

**Fig. 4:**
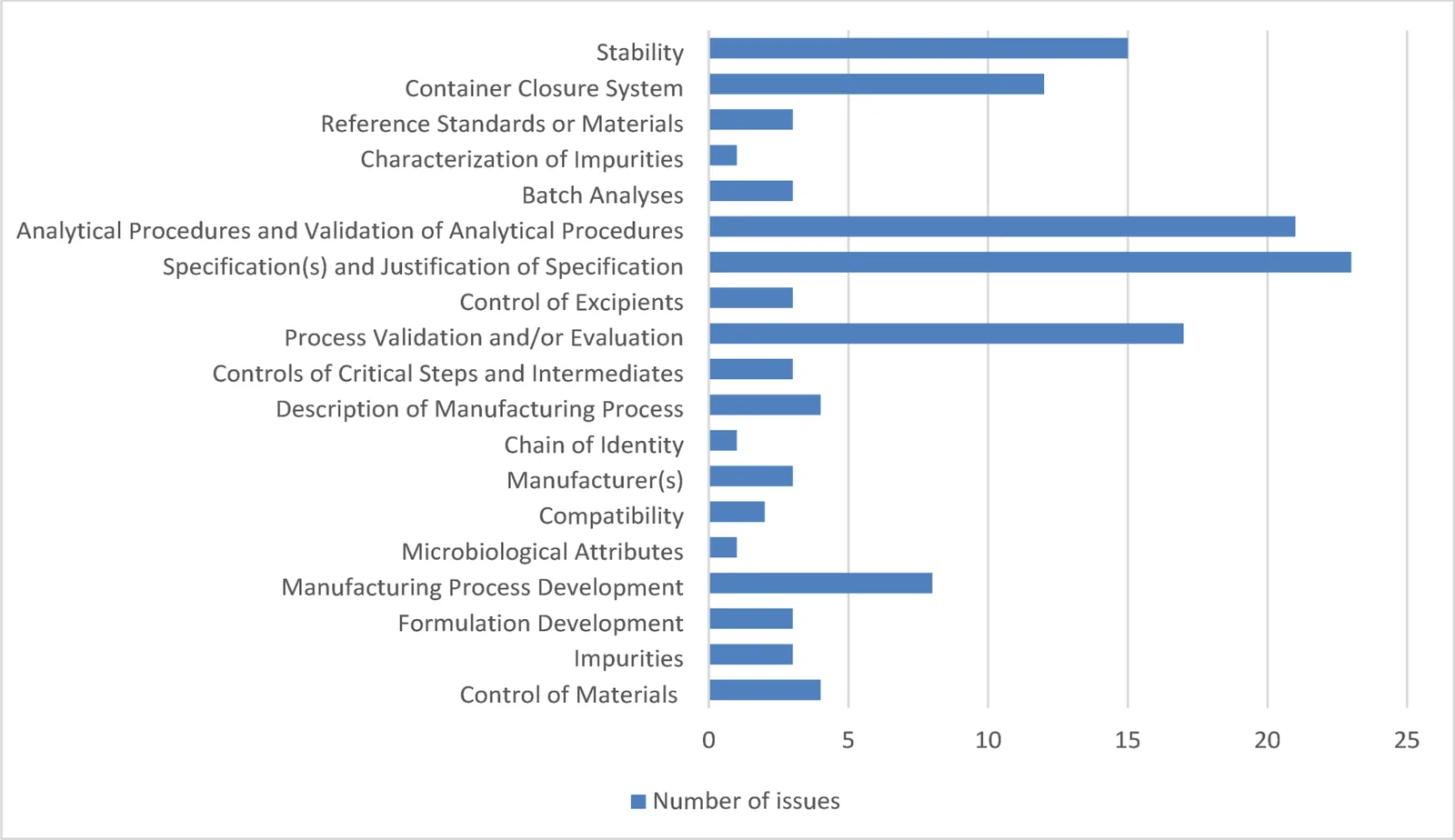
US quality review issues found in the biological license application (BLA) for approved cell gene therapies [1]. ^1^ Total number of concerns considering all applications analyzed.

The most common quality-related objections found in the EU MAA for the approved ATMPs, divided by “major issues” or “other concerns” are depicted in Fig. 3. The quality-related objections found in the US applications are depicted in Fig. 4. Details of the most common objections raised during the application review are summarized in Supplemental Table 1 (the same quality issue could be found for several products). For the EU, the most common “major” objections raised during the MAA review included comparability issues for manufacturing processes (19%) and issues with the potency assay (25%). The most common “other concerns” included comparability issues for manufacturing processes (17%) as well and issues with the stability data (17%). For the US, the process validation (13%), specifications and justification of specification (18%), analytical procedures and its validation (16%) and stability data (11.5%).

## Discussion

So far, several ATMPs have been approved in EU and the US, including cell gene therapies [10, 11].

The cell gene therapy field is moving fast; in oncology, CAR-T cell therapies have shown efficacy in treating certain hematological malignancies, such as relapsed/refractory B-cell lymphomas and leukemias, with some patients achieving long-term remission or even cure [12–14]. CAR T-cell therapies are advancing from last line of therapies to second line of treatments, where is expected that soon can reach first-line treatment option [15]. The first-ever CRISPR/Cas9 gene editing therapy was approved for market use (exagamglogene autotemcel; Casvegy) [16], the first-ever in-vivo mRNA CAR therapy for cancer [17] or the generation of CD19 CAR-T cells in situ is already being tested in clinical trials [17, 18]. TCR cell therapies are promising given that can target more cancers due to their ability of targeting tumor-specific sequences within a cell, and that is paving the way for next-generation therapies that tackle solid tumors [19, 20]. Hematopoietic stem cell-based gene therapies emerged as a lifelong reconstitution of the hematopoietic system with gene-corrected cells for monogenic diseases [21, 22], such as primary immune deficiencies and hemoglobinopathies. The first hematopoietic stem cell-based gene therapy and CAR-T therapy were approved in 2016 and 2017 respectively, completely shifting medicine from a chronic disease management approach towards potentially cure. However, it is important to note that long-term follow-up data are still being collected for many of these therapies to fully understand their durability and potential late effects [23].

### Regulatory Convergence

The EU and US ATMP framework includes different regulatory pathways to support bringing ATMPs through nonclinical and clinical development to MA. As cell and gene therapy innovation rapidly progresses, international efforts are underway to harmonize ATMP development and approval across regions, aiming to improve patient access. Regulatory agencies worldwide are increasing support for sponsors by launching new guidances, promoting international forum discussions, and working towards common agreements. Since 2020, the FDA has released or updated 17 guidances for ATMP development, covering regulatory considerations and focusing on specific products like CAR-T therapies and human genome editing of somatic cells [24]. The EMA has launched guidelines on overall manufacturing, nonclinical and clinical development, guidelines including checklists and flowcharts, and guidance on cell therapy labeling [25, 26]. These efforts reflect the growing experience and knowledge in the ATMP field, aiming to streamline the path from nonclinical and clinical development. Expedited development options in the EU and US are crucial for ATMPs targeting life-threatening or serious chronic diseases. Approximately half of EU PRIME or US RMAT designation requests for ATMPs are granted [10, 27], though there is room for improvement in eligibility criteria and agency resources [28, 29]. All approved ATMPs target rare conditions, with 43% addressing pediatric populations. This might add complexity to drug development, requiring deeper toxicology studies, specialized recruitment, pediatric-certified sites, developmental monitoring, and addressing unique safety and immunogenicity concerns [30]. Some of the analyzed products benefit from the Rare Pediatric Designation, incentivizing rare pediatric disease drug development, but the program is set to end in December 2024 [31–33]. New initiatives support rare disease drug development, including: (i) FDA’s Rare Disease Innovation hub [34]; (ii) support for clinical Trials Advancing Rare disease Therapeutics (START) Pilot Program [35] among other initiatives [36], (iii) potential UK regulatory pathway for ultra-rare disease therapies: Rare Therapies Launch Pad. These efforts aim to address the challenges in developing treatments for rare diseases that often lack commercial interest [37].

Dossier maturity is crucial for faster approval. In the EU, 6 out of 7 granted AA designations were reverted to standard timelines due to: (i) GMP inspection and certification requirements (*n* = 4), (ii) substantial issues and major objections (*n* = 4), and (iii) non-comprehensive data sets (*n* = 1). Related to the first point, most manufacturing sites are US-based, but EU regulations require a Manufacturer/Importer Authorization (MIA) for EU batch release and importation. A qualified person certifies batch release to ensure compliance with EU GMP standards [38]. For analyzed drugs, inspections occurred during MAA assessment. Regarding the second point, identifying development gaps through advisory procedures and addressing/justifying them in the dossier can reduce objections during review. PRIME and RMAT designations provide valuable support in this process. ATMP clinical development often relies on open-label, non-controlled studies rather than traditional Phase III trials, sometimes resulting in conditional approvals [39]. Requesting this authorization type upfront is an option; however, sponsors may be reluctant due to the post-marketing commitments. In the US, priority review aims to accelerate BLA assessment by 6–9 months. Rolling review allows concurrent submission and review of completed dossier sections, enabling quicker regulatory assessment and requests for additional information [40].

Although ATMPs are more frequently granted expedited development designations compared to other types of medicinal products, therefore benefiting from increased regulatory support and continuous interaction with the agencies, this advantage does not always appear to translate into higher-quality submissions. The relatively frequent identification of major quality deficiencies during the assessment of MAAs suggests that sponsors may not be fully leveraging the opportunities provided by such designations. In principle, the enhanced regulatory guidance associated with expedited pathways should enable the preparation of a more robust and comprehensive quality package at the time of MAA submission. This observation highlights the need for sponsors to optimize the use of these regulatory tools to ensure that accelerated approval and dossier completeness.

Multiple regional marketing applications can lead to redundant submissions, differing questions during assessment, and varied approval timelines. To enhance harmonization, the FDA and EMA plan to launch the Collaboration on Gene Therapies (CoGenT) Global Pilot. This initiative aims to explore concurrent submission and collaborative review of gene therapy applications with ICH regulatory partners, including Japan, Canada, and Switzerland, with potential expansion to align CMC and nonclinical issues earlier in development [41, 42]. Conditional approvals are more common in the EU, requiring additional post-market clinical data to confirm benefit-risk profiles. Interestingly, products with conditional MA in the EU often received standard MA in the US. While conditional MA represents a small fraction of overall centralized authorizations [43, 44], it applies to nearly half of approved ATMPs. The lack of robust trials may lead payers to restrict access or deny coverage, while further understanding of both short-term outcomes and long-term durability of gene therapies remains a challenge. Joint efforts with the EU Health Technology Assessments are also being done [45]. Despite reimbursement challenges, these therapies can be more sustainable for healthcare systems in the long term, particularly hematopoietic stem cell-based gene therapies with potential lifetime effects. These products are also becoming increasingly effective compared to standard care in oncology, targeting earlier treatment lines and potentially reducing costs associated with conventional therapy toxicity [46–48].

### Quality Development Optimization

Manufacturing complexity remains a major challenge in ATMP development, directly impacting clinical outcomes. Quality requirements maintain high standards regardless of the type of MA (standard, conditional, exceptional or accelerated approval). The impact of aspects such as comparability between different processes, scaling, potency, among others, on the final outcomes should be fully understood. Product quality attributes evaluation should follow quality risk management principles, addressing: identity, strength, potency, purity, process-related impurities, vector-related impurities, and other general attributes. A risk-based approach is recommended, evaluating: criticality of each quality attribute, severity of impact and uncertainty of clinical impact knowledge. Including quality results in a matrix format mapping relevant data and implemented mitigations for each identified risk in the MAA dossier is optional but may facilitate assessment and reduce objections during the procedure [49].

For the EU, the most frequent quality issues were related with manufacturing process comparability and potency assay. This finding aligns with the ones found by other authors; quality objections were mostly related to validation of the analytical methods (choice of the potency assays), design and control of the manufacturing process, and comparability [50]. For the US, the most common issues raised are related to the process validation, analytical procedures and its validation, specifications and their justifications and stability data.

The US usually requires detailed information on control materials and excipients, whereas nitrosamine impurities are a common focus in the EU. Both regions raised similar issues regarding manufacturing process development, requesting more robust demonstrations of comparability throughout development, with the US placing particular emphasis on process performance qualification. The FDA also requested modifications to commercial lot release specifications and clearer validation of analytical tests such as mycoplasma detection. In contrast, the EMA focused more on overall quality risk evaluation and the adequacy of the proposed release strategy, recommending re-evaluation of acceptance criteria based on post-approval data. The container closure system (CCS) was an important point in the US review, where the FDA focused on detailed testing, integrity, and leachables evaluation. Stability data were critically assessed in the US, with the FDA requesting additional long-term studies, protocol justifications, and alignment of shelf-life claims with supporting data. Conversely, the EU focused on confirming compliance with ICH guidelines and ensuring that stability data reflected commercial manufacturing conditions, with less emphasis on extensive additional studies. Overall, the US approach appears more data-driven and operational, requiring detailed documentation, validation, and immediate compliance with specifications, whereas the EU takes a broader, science-based view, emphasizing process comparability, justification of quality attributes, and continuous improvement.

For most autologous cell therapies, the manufacturing process from receipt of the apheresis starting material to final product is usually continuous, where the active substance immediately enters the finished product process, and no active substance is isolated. Therefore, it is acceptable that the specifications, analytical procedures, validation of analytical procedures, and batch analysis are only defined for the finished product.

The manufacturing process development and the comparability between processes is by far one of the major hurdles of cell therapy development. Comparability issues were raised during assessment of most products, and further data was required. Any alterations in material source, processing conditions, facilities or manufacturing steps might have changes in biological properties of the product, including both vector and cell manufacturing. To understand whether changes have impacted product quality attributes, and therefore potentially the clinical effect, a comparability assessment must be conducted. This assessment needs to cover the batches throughout clinical development and the proposed commercial manufacturing processes, or the site-to-site comparison when there is process transfer. This comparability exercise is usually based on analytical studies and not only on release specifications alone. If this is insufficient to determine the impact of the manufacturing changes on product quality, then nonclinical data might be required and clinical data as a worst-case scenario [51]. One of the challenges is establishing statistical relevance with limited lots [52]. The main comparability issues found during MAA assessments have been thoroughly reviewed by other authors [53]. For the analyzed products, the applicant provided further data with prospective and retrospective analysis and additional statistical analysis to support the comparability assessment, including justification for differences observed which were not expected to have a significant impact on the efficacy and safety of the final drug product. In other cases, this was not sufficient to address differences in batch release data from the different manufacturing processes; while not rendering the product non-approvable, the applicant had agreed on reconfirming and providing safety and efficacy updates post-MA. This is also consistent with previous findings, which observed that ATMPs tend to address certain quality issues during the post-approval phase, compared to other biologics [50]. There are two main available guidelines addressing the comparability expectations by regulators, the “*EMA Questions and answers Comparability considerations for ATMPs (2019)”* [54] and the FDA “*Draft guidance on Manufacturing Changes and Comparability for Human Cellular and Gene Therapy Products (2023)”* [55]. Having all the manufacturing history documented with tabular summaries of the process changes introduced during development as well as the rationales for changes is another point that might facilitate the application assessment. This might include vector, active substance and finished product analytical and functional comparability.

Assuring a product’s potency is another major hurdle of quality development and it is key for designing a manufacturing process and control strategy, the release of the product, product´s stability and comparability assessment [56]. Potency is the quantitative measure of biological activity, which ideally should be related to the clinical response [57]. For cell and gene therapies, viability and cell phenotype are important attributes; however, they are not sufficient on their own to fully characterize biological activity. Potency is also influenced by several key factors, including transgene or transgene protein expression, transduction efficiency, vector copy number per cell, and the percentage of vector-positive cells—particularly in hematopoietic stem cell-based gene therapy. In CAR cell therapies, potency is further linked to T-cell activation and cytokine release [58–60]. Potency assays are conducted under GMP conditions on final batches to ensure functional quality and consistency of the cell products. The current main guidelines for potency assays are the FDA “*Guidance for Industry Potency Tests for Cellular and Gene Therapy Products (2011)*” [61] and the Draft guidance, “*Potency Assurance for Cellular and Gene Therapy Products: Draft Guidance for Industry (December 2023)*” ^56^. So far, IFNγ assay is the accepted surrogate potency method for release of CAR-T therapies, to assess T cell activation and target cell killing (i.e., level of IFN-γ produced upon co-culture of anti-CD19 CAR T cells with CD19 + target cells). Cell viability and anti-CD19 CAR expression are included as well as part of potency testing for some products and part of identity and purity for others [62]. For the analyzed products, there were major issues with using tumor killing assay as potency test for release, which was replaced with the IFN-γ secretion assay as the commercial potency assay with the commitment to re-evaluate the specification limits for the assay following further manufacturing of commercial batches [63]. It is under discussion if the appropriate potency in vitro assay would need to simulate conditions of high tumor burden, induction of T cell exhaustion and provide readouts other than direct killing of target cells [64]. For the only approved TCR-cell therapy the accepted potency test for release is cytotoxicity assay with flow cytometry [65]. The current potency assays used for hematopoietic stem cell-based gene therapy with viral vector transduction include vector copy number, percentage of vector and gene positive cells, quantitative protein expression, and on-target editing frequency in the case of CRISP/Cas9 genome editing. However, theoretically the functional potency of transduced CD34 + cells would go beyond and the real potency would only be measured from clinical samples of the treated patients to measure engraftment capacity or functional expression of the transgene product [66].

There are also numerous challenges in validating drug product manufacturing processes and analytical methods. The major differences between process validation for autologous cell therapy and other products are the material availability constraints and high variability in starting material (i.e., patient cells with different disease state and affected by prior treatments). A common approach is to use surrogate cells from healthy donors as starting materials for process performance qualification (PPQ) batches and/or surplus patient apheresis material [38]. In some cases, the Agencies have not considered fully representative the use of a small-scale model with material from healthy donors for the full-scale performance, although it is considered acceptable as part of design of experiment studies (DoE) [63, 67, 68]. If surrogate cells are used, there is the need to demonstrate that is representative of drug product made from patient cells. This comparison can be made based on the data from developmental studies or based on manufacturing data available from a clinical studies [69]. Part of the PPQ studies also include container closure integrity testing studies to demonstrate the adequacy of CCS to maintain an effective barrier against potential contaminants. Deficiencies in extractables and leachables assessment was another common issue brought during review assessment. This risk-based assessment should evaluate the extractables and leachables in the manufacturing process and in the CCS to determine its extent to ensure patient safety. Depending on the study results, toxicological assessment might also be required. In the US, several applicants were required to conduct additional studies as post-marketing commitments for both container closure integrity and extractables/leachables testing. On the other hand, for most approved products, multiple interactions were held with the applicant during the review cycle to negotiate and refine batch release specifications and final acceptance criteria, leading to several amendments. For the analytical procedures and their validation, the applicant was committed as well to several post-marketing commitments, mostly related to the potency testing and the further validation of the sterility assay.

The current approved therapies are all autologous and centrally manufactured. However, to facilitate patient access timings to therapy, avoid hurdles in drug product distribution, having multiple manufacturing sites across different territories to meet demand, etc., the view is to shift towards allogenic cell gene therapies and decentralized manufacturing. It can take three weeks on average from the patient´s cell collection to treatment with a CAR T-cell therapy. With allogenic cells, a single source of cells to treat many patients makes it more scalable [70]. However, one main considerations for allogenic therapies, although is not expected to be common, is that the introduction of new donors will required an extensive characterization and comparability assessment, which also might generate hurdles [53]. Avoiding the immunological responses of these therapies is also one of the major current hurdles to overcome [71].

The closed and automated cell therapy market is experiencing rapid growth, and the benefits from regulatory standpoint include reducing contamination risks and ensuring safety, faster processing times, while automation helps in maintaining compliance with GMP. Decentralised manufacturing has not used so far in any of the authorised ATMPs. While the regulatory pathway for decentralized manufacturing is being debated now, manufacture cell therapies in the same site as the patient, might make a difference for improving patient access to autologous treatments. The challenge is ensuring that decentralized manufacturing units are compliant with regulatory requirements for GMP as well as demonstrate that the manufacturing in multiple in sites, does not adversely affected product quality, safety or efficiency. The Quality and Innovation Group at the EMA is currently working on developing a Q&A guideline to address how to demonstrate “comparability” between all decentralised sites in equivalent manner to a centralized manufacturing [72]. How to perform this comparability exercise, how to keep the process comparable, how to validate every new site, the control of variability of raw materials at every site, etc. are uncertain key points. From regulatory perspective, how to manage the GMP inspections, the maintenance of many decentralised dossiers or how to ensure a qualified person would oversight the operations and release are also points under consideration [38, 73, 74]. Some sponsors are already producing products through decentralized cell therapy manufacturing platform [75, 76].

Finally, new designations to support manufacturing are also being launched, such as the FDA’s Advanced Manufacturing Technologies Designation Program, for manufacturing processes that imply novel technologies [77].

### Limitations of the Study

The study analysis relies on publicly available data. However, due to intellectual property protections and confidentiality, the MAA or BLA dossiers, regulatory agencies’ findings, and the outcomes of discussions during the application review process are not fully disclosed, limiting the analysis.

Another key limitation of this study lies in the differences in public disclosure policies between the EMA and the FDA regarding the quality sections of marketing authorization dossiers. While the EMA publishes EPARs that include structured summaries of manufacturing processes, comparability data, and control strategies, the FDA provides only limited quality-related information through its publicly available Summary Review Documents. Consequently, the greater level of detail available in EU sources compared with US documents may introduce a regional bias, as the analysis relies on more publicly accessible EMA data.

### Recommendations and Future Perspectives

Based on lessons learned from previous experiences with the development and approval of cell gene therapies, developers should engage early and frequently with regulatory agencies through available designations such as PRIME (EU) and RMAT (US) to proactively address potential issues and increase approval success. A strong focus on developing and validate the potency assays as early as possible in the development process is crucial. Developers must prioritize manufacturing process comparability studies throughout development to ensure comprehensive data is available for regulatory submission. Additionally, planning GMP inspections well in advance, to ensure compliance with requirements for batch release and importation in EU, particularly for ex-EU manufacturing sites, which might prevent EU approval delays. Investing in comprehensive stability studies and providing a well-justified rationale for specifications is essential. For paediatric indications, developers should consider specialized toxicology studies and early recruitment strategies to facilitate trial success and development timing feasibility. It is advisable to leverage new regulatory initiatives such as the FDA’s Rare Disease Innovation Hub and the START Pilot Program for rare disease therapies. Preparing a mature, high-quality dossier that addresses potential gaps identified through advisory procedures can streamline the review process. Additionally, it is essential to develop a clear strategy for addressing post-marketing commitments, particularly for conditional/accelerate approvals. These recommendations aim to address common challenges and optimize the development and approval process for cell and gene therapies based on regulatory experiences to date.

The field of cell gene therapies is expected to undergo significant advancements in the coming years. Key areas of development include several promising strategies: (i) allogenic “off-the-shelf” and in vivo CAR-T cell products that overcome manufacturing challenges and improve accessibility [18, 78], (ii) in vivo gene editing and CRISPR-based therapies, delivered directly to patients, showing great potential for treating genetic disorders [79], (iii) next-generation CAR-T and TCR therapies, designed to address solid tumors more effectively [80], (iv) non-viral delivery methods, such as lipid nanoparticles, which may enhance safety and streamline manufacturing [81], (v) combination therapies that integrate cell therapies with other modalities, such as checkpoint inhibitors, to improve efficacy [82], and artificial intelligence in manufacturing, which could optimize production processes and reduce costs [83]. These innovations aim to expand the therapeutic potential of cell and gene therapies while addressing current challenges related to efficacy, safety, and accessibility.

## Conclusions

This article provides key insights for developers to anticipate potential challenges during the approval review process from both a quality and regulatory perspective. The possibility of offering curative treatments to patients who previously faced a fatal prognosis has generated significant expectations among clinicians. However, the complexity of ATMP development, particularly for rare diseases, presents multiple hurdles, including manufacturing challenges, evolving regulatory standards, and post-commercial barriers that can impact both market entry and long-term sustainability. Emerging therapeutic modalities, such as CRISPR/Cas9-based therapies and TCR-T cell therapies, along with new indications like autoimmune diseases, introduce additional complexities beyond those currently encountered. To overcome these challenges successfully, sponsors must adopt an integrated approach from early development, ensuring a holistic strategy that addresses regulatory, manufacturing, clinical, and healthcare system integration challenges. This proactive approach is essential to securing both regulatory approval and long-term commercial viability.

In conclusion, while regulatory frameworks in both the EU and the US have evolved to support the rapid development of cell and gene therapies, quality development remains the critical determinant of regulatory success. The most common deficiencies identified - comparability between manufacturing processes, potency assay validation, and stability data - highlight the need for earlier and more systematic integration of quality considerations throughout development. Strengthening dossier maturity, supported by proactive regulatory interactions through available regulatory designations, can substantially reduce objections during review and accelerate approval.

## Electronic Supplementary Material

Below is the link to the electronic supplementary material.

Supplementary Material 1.

## Data Availability

No datasets were generated or analysed during the current study.
