# Supplementary material for: Outlook of Cell Gene Therapies Development and Approval from Quality and Regulatory Perspective
Source: Ther Innov Regul Sci. 2026 Feb 12;60(3):823–36. doi: 10.1007/s43441-026-00920-4 (PMC13110239; doi:10.1007/s43441-026-00920-4)
Supplement: Supplementary file 1 — Supplementary Material 1. [file 43441_2026_920_MOESM1_ESM.docx]

# Supplemental Tables

## Supplemental Table 1. Quality related objections found during MAA/BLA for approved cell gene therapies

| Quality topic | Region | Quality issue |
| --- | --- | --- |
| Control materials | US | - Incomplete information to assess if adequate procedures are in place to control raw materials and leukapheresis material - Requested specifications for all inert materials - Provide justification of specifications and documents for all raw materials - Provide a description of the qualification audit, the requirements for the collection, labeling, packaging, shipping, and documentation of the leukapheresis material and the training provided and indicate how often each facility undergoes requalification |
|  | EU | - Improving control of raw materials as recommendations for future quality development |
| Control of excipients | US | - Clarifications on testing procedures and validation of analytical procedure for CryoStor® CS10 - Cryoserv® (≥99.0% Dimethyl Sulfoxide solution) is an excipient that is not included in a major pharmacopoeia as an injectable solution. The information in the original submission was inadequate for a full review, additional information requested - Further data requested to support safety of the from human or animal derived adventitious agents |
| Impurities | US | - Safety margin was calculated for each impurity (Exposure Limit/Impurity level in a single dose) assuming no clearance. Additional details on the limits identified from the original publications and any adjustment factors applied requested - Provide additional data supporting clearance of the anti-CD3 and anti-CD28 antibodies - Residual Cas9 and sgRNA were not assessed |
|  | EU | - Specification, potency and impurities acceptance criteria for bulk and sterile fill sgRNA - Detected a NDMA nitrosamine impurity in the CryoStor CS5 excipient |
| Formulation Development | US | - The original formulation process allowed for an excessive volume of the DP components above the intended dose to be delivered to the clinical site - T cell concentration clarifications |
| Manufacturing Process Development | US | - Further information requested on the process characterization studies that analyzed the product CQAs at each process step - Optimization of filling scheme by expanding target fill volumes - Clarifications related to shipping form requested - No data supporting post-thaw/in-use stability of DP were provided. Required as PMC - Requested to provide data supporting the combination of healthy donors and patient material for comparability analysis - Additional data requested on comparability for the formulations used in clinical studies - Requested data supporting comparability of the small-scale and full-scale manufacturing runs, including data on unit operations - Requested to analyze CMC data by including only lots manufactured with the current manufacturing method and asked update release criteria to reflect data obtained by lots reflective of the manufacturing process. - Issues with comparability of the manufacturing process DP used in clinical/commercial lots |
|  | EU | - Insufficient demonstration of comparability of different manufacturing processes (clinical and commercial processes) - Insufficient demonstration of comparability for clinical batches manufactured at different sites - Differences for some CQAs and the low number of batches used for the comparability exercise - Final product comparability for the different sites and vector starting materials could not be conclusively demonstrated - Insufficient information and data confirming the suitability of the transduction unit operation - Concerns on the process control strategy for transduction efficiency - Provide further justifications for the limits proposed for each of the CQA - Provide additional information on process parameters and their classification and the justification of the proposed ranges - Proposed to revise the acceptance limits or provide further justification to maintain the proposed criteria for potency attributes (risk of sub-potent batches) - Differences of quality attributes between US vs EU studies batches - Justify the range of VCN in transduced cells - Improvements are required to increase for CD34+ yield as recommendation for future development - The lower side of the PAR for the post electroporation duration is not considered acceptable (may vary for batch with lower editing efficiency) |
| Microbiological Attributes | US | - Bag defect observed after simulated transport, concerns with container closure integrity in terms of protection from microbial ingress |
|  | EU | - Deficiencies in the microbiological control strategy during manufacturing procedure |
| Compatibility | US | - The pore sizes of the filters that can be used were given as a range, but only compatibility data for certain blood filter was provided - Clarifications to support compatibility of cell suspension in container closure system |
| Manufacturer(s) | US | - Requested to provide information regarding testing performed at each facility - Insufficient information to support assay use at the testing site, as the product-specific qualification was only performed at the US site and applicant did not provide data to support assay use at testing site. |
|  | EU | - Final product manufacturing site in the USA. Lack of valid EU GMP certificate |
| Chain of Identity | US | - Concerns to ensure that a hybrid system is able to maintain the integrity of production data and the COI of each autologous DP lot - Clarifications for the procedures to maintain COI/COC for the duration of product storage and information on qualifications of authorized treatment centers. |
| Controls of Critical Steps and Intermediates | US | - Further clarifications on normal operation range and PAR for hold time in cryopreservation media - The CPP ranges are supported by manufacturing experience. However, several media components are provided as targets and not ranges |
| Process Validation and/or Evaluation | US | - The PPQ summary provided in the original BLA submission was not the full PPQ report and did not include additional in-process testing results - The leukapheresis collection volume was based on shipping validation. However, the lower limit is below the PPQ and clinical experience - Not evaluated some of the high-risk process components, which may result in underestimation of the leachables profile in the final product - The original submission did not provide adequate information for review - Additional clarifications requested for the deviations encountered during the PPQ runs - Based on the results of this capacity test, the performance of culture initiation and PBMC isolations is not supported - Operational qualification report for shipping missing in the BLA submission and no data on other DP CQAs pre- or post-transport to further support that the shipping conditions do not have adverse effects on product quality - The transportation qualification study was not sufficient to demonstrate that the process is adequately controlled - Shipping validation studies would not evaluate the effect on the DP CQAs - Shipping validation was limited to one lot which was not manufactured and tested using the commercial process and facility - Evaluation of the extractables and leachables studies determined that the study was inadequate to address elemental leachables and cumulative leachables in the DP through processing and storage |
|  | EU | - The use of PARs as process validation acceptance criteria for the critical process parameters in the process performance qualification runs has not been justified |
| Specification(s) and Justification of Specification(s) | US | - DP commercial lot release specifications and justification of specifications requested to be modified during procedure. The commercial specifications would also be applied to the stability testing program. - Proposed acceptance criteria not supported by clinical experience. During the review cycle, acceptance criteria modified from those originally submitted to better reflect clinical study and manufacturing experience. - Analysis of the percentage of T cells and potency test data indicates the lots meet the proposed acceptance criteria cells, but the analysis also supports ability to set higher acceptance criteria - Provide additional information on the lots that were excluded from the statistical analysis used to set the acceptance criteria - Insufficient data had been provided to support the comparability of the mycoplasma test - The mycoplasma test was not performed on the proper sample. Generally, mycoplasma testing is performed on the cells and supernatant to increase assay sensitivity. The assay was not validated in the proper matrix - Cell concentration performed as part of dose determination - acceptance criteria initially proposed only used lots manufactured with a comparable process, which is not the proposed commercial facility - Requested to include additional sampling time points of the DP for some specifications such as total cell concentration to confirm on the final formulated DP - Opacity - lots with cell clumps and other foreign matters should not be released based on visual inspection. |
|  | EU | - Lack of a risk evaluation for the potential presence of nitrosamine impurities - Re-evaluation of the release tests and their acceptance criteria based on post approval data - Concern on applicant’s initially proposed release strategy as additional controls would be required to justify the proposal from a quality perspective |
| Analytical Procedures and Validation of Analytical Procedures | US | - Sterility assay was not considered to be properly validated - Missing several SOPs not provided in the BLA - Gentamicin validation comparability issues - No data was provided on the repeatability/intermediate precision of the cell viability assay - Assay validation acceptance criteria for the Anti-CD19 CAR T cell assay validation study was not provided - Validation of the anti-CD19 CAR expression not provided - Acceptance criteria for some assays considered to be relatively broad. Using such wide criteria may lead to acceptance of higher assay variability - Qualification of assay standards for RCL. Co-validation of the RCL assay was unsuccessful at different sites; until successful assay validation is performed at these sites and laboratory equivalence between sites is demonstrated this assay may only be performed for commercial lot release testing at one site - Additional information requested, e.g., information on training used to qualify operators for appearance test for DP release and stability, instrument to instrument variability, etc. - Further studies are required to address assays for release testing - Identity, Purity, Viability Validation of Analytical Procedures - differences between results obtained from clinical DP lots and the validated assay ranges - Clarifications requested for appearance, potency, Identity, Purity, Viability, Cell concentrations methods - Additional information regarding robustness testing of the VCN assay - Change in the CAR-T test identity during development, data comparing CAR expression results from those obtained during PPQ studies and those obtained during assay validation studies for the same lots had to be provided - Clarifications requested for the acceptance criteria for system suitability for the potency assay - Extensive interactions held during the review cycle for the potency assay resulting in replacement of the assay |
|  | EU | - Disagreement with the suitability of the proposed potency test - Disagreement with the proposed acceptance criterion for the potency assay - Provide method description and validation of microbiological control - Request for data demonstrating the successful transfer of the potency assay to the batch release testing site |
| Batch analysis | US | - Additional information regarding the lots that failed appearance testing - Clarifications related to lot failure rate. Proposed implement measures to reduce this failure rate. |
| Reference Standards or Materials | US | - Positive control (QC) CAR T cells for CAR expression - additional details regarding generation and qualification of assay standards and controls - Further justifications on acceptance criteria for a positive control lot |
|  | EU | - Additional information on the manufacture and qualification for each of the reference materials |
| Container Closure System | US | - Additional information regarding bag acceptable quality level and descriptions of the shipping system and packout/unpack procedures - The applicant did not conduct testing on the bag to demonstrate the bag can withstand drop events and extreme temperature conditions. This testing is being requested in a PMC - The applicant provided insufficient information on the extractables and leachable chemical profile of the bag. This testing is being requested in a PMR - The primary packaging components are not derived from animal sources, and the applicant did not provide details TSE risk - Additional information requested on container closure system bag kit - Deficiencies found in the study of CCI of the Cryogenic Storage Container - Further clarifications requested on concentration of leachables - Extractable and leachables study is not adequate. The study represents accelerated study for storage and in use-hold and may underestimate the leachables due to temperature stress of the bag comparing to actual conditions (e.g., -130°C). Accelerated conditions can be used in addition, but not instead of real-time study. The study also did not assess leachables originating from the high-risk process steps - The release for infusion (RFI) certificate was requested |
|  | EU | - Only limited time point data is available from the oxygen head space method for CCI tests, 12 and 24-month timepoints are required |
| Stability data | US | - Justification for use of stability protocol acceptance criteria that differed from the commercial acceptance criteria - Additional patient lots should continue to be studied for stability (post-approval stability protocol) - Data submitted in the original submission and in amendment was not sufficient to support the proposed long-term shelf-life - Provide justification for the time points for which stability testing was omitted - Provide data to support T cell percentage in the DP - The lots used for in-use stability studies were healthy donor-derived and filled into bags rather than being patient-derived material filled into the commercial container-closure. - Additional long-term stability data requested - Cryopreservation bag leachables study was conducted for only 12 months. The shelf-life for DP cannot exceed that supported by the leachable study until final study has been submitted and reviewed - Ongoing long-term maintenance of sterility studies use the commercial container closure, If possible, this material should also be tested for potency, viability and at the scheduled time points - Clinical stability lots were manufactured at X site rather than the commercial manufacturing site |
|  | EU | - Availability of data to support the shelf-life claim in line with the requirements of ICH Q1A-R2. - Stability data from patient derived material with respect to viability are not available - During the procedure all available data of the stability program have been requested to confirm the data with batches of the commercial manufacturing process - Providing additional stability data for starting materials as recommendation for future quality development |

CCI: container closure integrity; COI/COC: custody of identity; CPP: critical process parameters; CQAs: critical Quality Attributes; DP: Drug Product; PAR: proven acceptable ranges; PMC: post-marketing commitment; PMR: post-marketing request; PPQ: process performance qualification; SOPs: standard operating procedures; TSE: [transmissible spongiform encephalopathies](https://www.fsai.ie/enforcement-and-legislation/legislation/food-legislation/meat-fresh-meat/transmissible-spongiform-encephalopathies-(tse)-bo); RCL: replication Competent Lentivirus; VCN: vector copy number.
